# National Insurance and Unqualified Practitioners

**Published:** 1913-05-03

**Authors:** 


### The Hospital

The Workers' Newspaper of Administrative Medicine and Institutional Life, Administration, National Insurance and Health.

No. 1400, Vol. LIV. SATURDAY, MAY 3, 1913. PRICE ONE PENNY.

### NATIONAL INSURANCE AND UNQUALIFIED PRACTITIONERS.

The letter which appeared in our columns on April 19 (p. 79) from the General Secretary of the National Medical Guild revealed certain facts concerning the recognition of unqualified medical practice by Insurance Committees which certainly deserve more than casual attention. It appears that, with the knowledge and sanction of the Commissioners, various Insurance Committees are permitting insured persons to "make their own arrangements for medical benefit" with herbalists and other unqualified practitioners. The Guild decided to probe the matter fully, and elicited a statement from the Commissioners to the effect that there is nothing in the Act or in the regulations as to medical benefit which specifically forbids this to be done; and a question asked in Parliament was answered in exactly the same sense. It is therefore evident that the State is in effect endorsing unqualified medical practice, is recognising it and subsidising it officially.

The unfortunate taint of party politics and caucus tactics hangs heavy in the whole atmosphere of National Insurance. For reasons of political expedience it was thought necessary to make the Insurance Act a party affair, and to rush it through Parliament without that proper discussion which it admittedly ought to have had. Without discussing now to which party the major blame attaches for this anti-social and anti-progressive procedure, it suffices that the disastrous policy thus initiated pervaded thenceforward everything connected with the working of the Act. Dissatisfied with the terms offered them, the doctors revolted against service under the regulations, and were promptly treated as political enemies to be outmanœuvred by all the subterfuges and stratagems of political warfare, in which notoriously all is fair. Beaten they were, and badly, but by no means finally crushed; and it may be well that this episode of unqualified practice is merely another move in the game designed to bring them finally to heel. We should hesitate to suggest or to credit such an idea, had we not the experience of the last six months to assure us that the engineers of the party machine are far more concerned to secure its progress than that of the nation at large. The Commissioners can stop such arrangements by the Committees if they think fit; and if they do not do so, they are parties to the official recognition of unqualified practice.

When the defeat of the British Medical Association on the question of remuneration for "panel" service occurred at the beginning of this year, large numbers of insured persons in certain districts and certain occupations found themselves faced with the prospect of what they regarded as very inferior medical attendance to that which they had been in the habit of being able to command. Some thousands of them, to avert this undesired consequence, exercised their right under the Act to apply for permission to make their own medical arrangements. At the time this happened not to suit the exigencies of the political situation, so every possible obstacle was thrown in the way of these people by the Insurance Committees, in the interests of the Ministerialist party. The machinery available for the purpose was set in motion quite remorselessly, and, with inconsiderable exceptions, the recalcitrant insured were coerced into accepting the panel system. Now, presumably, it happens not to suit the calculations of the moment to continue this policy in certain districts. Permission is being granted to insured persons, therefore, to make their own arrangements with herbalists, Christian Scientists, and quacks of all sorts; and the plan has the additional merit of serving to put another turn of the screw on to the medical profession. Apparently unqualified practitioners have not yet been accepted on the panels: that perhaps would be too much for even politicians to swallow. But this more subtle method is probably acceptable to the authorities as an additional hint to the medical profession that entire subservience is the only basis upon which it will henceforth be permitted to exist.

If the medical benefits of the Act were really, as the country was originally led to believe, inclusive of all and every kind of treatment necessary in sickness, it would be arguable that those who were foolish enough to make their own arrangements with herbalists should be left to their fate. But medical benefit, as now evolved, is only for the minor ills of life (tuberculosis excepted); and the voluntary system remains as much a necessity as ever to the bulk of the people overtaken by serious sickness. No institution would, of course, refuse an otherwise eligible patient just because he had placed himself under unqualified care; but if the patient were unable to attend the hospital in person delay would inevitably ensue. So, too, in regard to consultations and anæsthetics. No qualified practitioner can assist an unqualified one in either capacity; and thus second opinions and anæsthesia for minor operations are equally denied to the patient who accepts unqualified service. The Commissioners know all this, if the insured do not; theirs is the responsibility, and to attempt to saddle it on the Committees would be unworthy of the important office they hold.

